# Measuring serum estradiol and progesterone one day prior to frozen embryo transfer improves live birth rates

**DOI:** 10.1186/s40738-020-00075-2

**Published:** 2020-04-14

**Authors:** Snigdha Alur-Gupta, Margaret Hopeman, Dara S. Berger, Kurt T. Barnhart, Suneeta Senapati, Clarisa Gracia

**Affiliations:** 1 Division of Reproductive Endocrinology and Infertility, University of Pennsylvania, Philadelphia, Pennsylvania USA; 2 Present address: Center for Reproductive Medicine & Advanced Reproductive Technologies, Twin Cities, Minnesota USA

**Keywords:** Assisted reproductive technology, Frozen embryo transfer, Hormone surveillance, Live birth, Pregnancy, Progesterone level

## Abstract

**Background:**

Given no consensus in the literature, this study sought to determine if a protocol of measuring serum estradiol and progesterone the day prior to frozen embryo transfer (FET) improves likelihood of pregnancy and livebirth.

**Methods:**

This was a retrospective time-series study of women undergoing autologous vitrified-warmed blastocyst programmed FETs at an academic institution. Live birth rates were compared between a surveillance protocol, where serum estrogen and progesterone surveillance are performed the day prior to a programmed FET, and a standard protocol, whereby no hormonal lab evaluation is performed the day prior.

**Results:**

Three hundred seventy-nine standard FET and 524 surveillance FET cycles were performed. Patients in the surveillance protocol were significantly more likely to achieve live birth (51% vs. 39%; aOR 1.6, 95%CI [1.2, 2.2]). Obese women were noted to be more likely to have lower progesterone hormone levels on surveillance labs (OR 3.2, 95%CI [2.0, 5.3]). However those whose hormonal medication dose was modified because of pre-transfer labs were as likely to achieve live birth as those whose dose was not modified (47% vs. 53%; aOR 0.8, 95%CI [0.6, 1.2]).

**Conclusions:**

Cycles with the surveillance protocol were more likely to result in live birth. Patients with low levels of pre-transfer hormones, such as obese patients, likely have lower pregnancy rates. It is possible that when these levels were corrected after measurement, pregnancy rates improved to match those whose levels were not low enough to warrant intervention.

## Background

Frozen embryo transfers (FETs) have increased in prevalence, with autologous frozen embryo cycles accounting for 32.7% of all ART cycles conducted in the United States in 2016 [1]. FETs are performed not only to utilize supernumerary embryos but are recommended for women with an inadequate endometrium, or those pursuing preimplantation genetic testing (PGT) [2, 3]. As use increases, it is important to identify factors that improve outcomes [4]. Several known predictors include age and number and quality of frozen embryos [4]. Another vital aspect is the ‘window of implantation’, the time period when the endometrium is most receptive [3, 5]. This state is induced through an interplay of hormones including estrogen and progesterone [6]. Prior evidence has shown that premature elevations in progesterone alter this window and can be associated with lower pregnancy rates in fresh cycles [7–9].

Therefore, when performing FETs an attempt is made to replicate the natural hormonal environment. While extensive research has evaluated ideal protocols for endometrial preparation, no definitive method has been established [10–12]. The optimal route of progesterone or estradiol administration [13–15] and duration of progesterone administration [16, 17] have all been studied. While intramuscular progesterone has been shown to be superior to Endometrin for ongoing pregnancy rate in an interim analysis of a large randomized non-inferiority study [15], significant differences in implantation, clinical pregnancy and live birth rates were not observed in a retrospective evaluation comparing vaginal progesterone gel to intramuscular progesterone [14]. It is also unclear whether hormone levels surrounding time of frozen transfer are associated with outcome. In a review of women undergoing autologous euploid embryo transfers with intramuscular progesterone supplementation, day 19 progesterone levels above 20 ng/mL were noted to be associated with lower live birth rates, with more drastic decreases as progesterone levels crossed 40 ng/ml [18]. However a separate study assessing fresh untested day 3 donor embryo transfers with intramuscular progesterone supplementation found that progesterone levels above 20 ng/mL were instead associated with higher live birth rates [19]. A study of autologous euploid frozen embryo transfers with vaginal progesterone support found that women in the lower quartiles of serum progesterone levels (< 10.64 ng/mL) the day prior to transfer had significantly higher miscarriage and lower live birth rates compared to those in the other quartiles [20]. A similarly structured prospective cohort study of both fresh and frozen donor oocyte transfer cycles with vaginal progesterone support found that women with progesterone levels under 9.2 ng/mL had significantly lower adjusted ongoing pregnancy rates compared to those with levels above this threshold [21]. However, both these studies used vaginal progesterone supplementation which was shown to be inferior as described above and makes interpretation of serum levels more difficult. The varying patient populations, use of autologous versus donor oocytes, evaluation of both fresh and frozen cycles and differing routes of progesterone administration in all of these studies make it challenging to draw conclusions.

Given the conflicting information and the biologic plausibility by which these hormonal measurements are important, we sought to study whether a protocol of monitoring pre-transfer estradiol and progesterone levels and adjusting hormone doses impacted pregnancy and live birth rates in a well-defined modern cohort of women undergoing autologous frozen embryo transfers with intramuscular progesterone supplementation.

## Material and methods

This was a single center retrospective time-series study conducted from January 2013–February 2017. Reproductive aged women undergoing programmed autologous vitrified-warmed blastocyst transfers, referred to as FETs, were included. Our laboratory uses vitrification and thaw media manufactured by Fuji Film Irvine Scientific (Santa Ana, CA). Embryos are frozen and stored on CBS High Security Vitrification straws manufactured by Cryo Bio System (L’Aigle, France). We converted from a slow freezing protocol to vitrification during the third quarter of 2012, before this study inclusion period. Some of the earlier embryos may have been frozen by the original slow freezing protocol, however still thawed using Irvine Scientific media, per laboratory protocol. Those using donor oocytes, gestational carriers or natural cycle FETs were excluded. Patients of all ages and fertility diagnoses were included. Patients who had FETs performed between January 2013 and June 2015 received the standard FET protocol, described in further detail below and referred to as the “standard protocol”. Beginning in June 2015, serum estradiol and progesterone levels were obtained the day prior to transfer in order to determine if hormonal medication dose adjustments were required prior to the FET. Those who had FETs performed after June 2015 thus received the standard protocol with the addition of the pre-transfer lab surveillance, referred to as the “surveillance protocol”, reviewed below.

### Standard protocol

A FET was performed by first administering luteal phase GnRH agonist suppression for a minimum of 12 days. Ovarian suppression was confirmed with baseline hormonal and transvaginal ultrasound assessment. If confirmed to be suppressed, oral estradiol was initiated at a dose of 2 mg and titrated to 6 mg daily over the course of 12 days. Transvaginal ultrasound and bloodwork was performed after these 12 days and embryo transfer was scheduled if the endometrial thickness was at least 7 mm and of a trilaminar morphology and estradiol levels at least 200 pg/mL. If progesterone levels were greater than or equal to 1.5 ng/ml, the cycle was cancelled with plan for higher doses of GnRH agonist in subsequent cycles. In cases of either inadequate estradiol level, endometrial thickness or morphology, typically vaginal estradiol at a dose of 1-2 mg or higher doses of oral estradiol (increased by increments of 2 mg) were administered for approximately 1 week and bloodwork and transvaginal ultrasound were repeated to assess if above specified criteria were met. Intramuscular progesterone was initiated at 50 mg nightly when parameters were met, and blastocyst transfer was scheduled to occur on the 6th day of progesterone supplementation.

### Surveillance protocol

Endometrial preparation followed the same methods as outlined above. However, patients undergoing FETs after June 2015 also had serum estradiol and progesterone surveillance the day prior to their FET, on the 5th day of progesterone supplementation. Estradiol doses were increased by 1–2 mg vaginally or orally by 2 mg for levels below 150 pg/mL. Progesterone was typically increased from 50 mg to 75 mg IM nightly, if levels were below 15 ng/mL. These thresholds were established based upon clinical consensus within the center, given that there are no uniform threshold recommendations based upon prior research. Transfers in both protocols were cancelled for inadequate endometrial lining or morphology or inappropriate estradiol levels.

### Outcome assessment

Serum hCG was performed 10–12 days following transfer. bHCGs > 1 mIU/mL were considered positive and used to define pregnancy rate. Primary outcomes were pregnancy rate and live birth rates. The following definitions were employed for secondary outcomes: implantation rate: number of fetal sacs divided by number of embryos transferred, biochemical pregnancy: positive hCG that spontaneously dropped to < 1 mIU/mL in the absence of an intrauterine gestational sac; clinical intrauterine pregnancy: presence of an intrauterine gestational and yolk sac on ultrasound; spontaneous abortion: loss of clinical intrauterine pregnancy; therapeutic abortion: induced loss of clinical intrauterine pregnancy and stillbirth: pregnancy losses at greater than 20 weeks gestation.

### Analysis

In order to have 80% power to detect 10% increase in pregnancy rates between the two groups, 380 cycles per group were needed. Categorical data was analyzed using chi-square test whereas continuous data was analyzed by either student t-test or Wilcoxson ranksum as indicated by normality assessment. Multivariate logistic and linear regression was used to compare primary and secondary outcomes, adjusting for age, body mass index (BMI), usage of PGT, and number of embryos transferred. Confounders were determined *apriori* as well as through a backwards elimination strategy.

Multivariable analysis was first conducted to compare primary and second outcomes for women in the surveillance protocol versus those in the standard protocol. In a sub-analysis, women in the surveillance protocol who had hormonal medication dose adjustment as a result of surveillance labs were then compared to those in the surveillance protocol whose doses did not require adjustment. Sensitivity analyses were conducted to evaluate differences in live birth rates between the surveillance and standard protocols for those who had freeze-all cycles (defined as those who did not have a fresh embryo transfer at the time of stimulation) and those who did and did not have PGT. These specific groups were chosen as patients with freeze-all cycles and PGT are thought to be better prognosis patients which could influence primary outcome results. Analysis was performed using STATA v.14.

## Results

### Baseline characteristics: surveillance versus standard protocol

Three hundred seventy-nine FETs were performed with the standard protocol and 524 were performed with the surveillance protocol. Cancellations, which occurred in 4.1% of standard and 5.8% of surveillance protocol cycles, were most often due to structural factors such as thin endometrial lining (< 7 mm). The mean age and BMI of women did not differ significantly in the two groups. Those in the standard protocol group were significantly less likely to have PGT and more likely to have more embryos transferred and have multiple gestations (defined by number of heartbeats) (*p* < 0.001). (See Table 1).

**Table 1:** Baseline Characteristics of Patients in Surveillance vs Standard Protocols

	Surveillance protocol (***N*** = 524)	Standard protocol (***N*** = 379)	***p***-value
**Mean Age (years)**	34.6 (+ 4.3)	34.7 (+ 3.9)	0.7
**Mean BMI (kg/m**^**2**^**)**	25.3 (+ 5.3)	25.0 (+ 5.3)	0.13
**Infertility Diagnosis (%)**	Unexplained – (25%)	Unexplained – (23%)	0.1
	DOR – (6%)	DOR – (8%)	
	Endometriosis – (3%)	Endometriosis – (8%)	
	Ovulation disorders – (11%)	Ovulation disorders – (10%)	
	Male factor – (21%)	Male factor – (19%)	
	Other^a^ – (34%)	Other^a^ – (32%)	
**PGT testing of embryo, n (%)**	167/524 (32%)	52/379 (14%)	< 0.001
**Number of embryos transferred per cycle, n (%)**	1–351 (67%)	1–194 (51%)	< 0.001
	2–168 (32%)	2–174 (46%)	
	3–5 (1%)	3–10 (3%)	
**Number of heartbeats, n (%)**	1–245 (76%)	1–135 (80%)	< 0.001
	2–41 (13%)	2–33 (19%)	
	3–4 (1%)	3–0 (0%)	

^a^ = Included patients with tubal factor, uterine factor and other as infertility diagnoses

### Surveillance versus standard protocol

In multivariable logistic regression analyses, patients undergoing FETs with the surveillance protocol were significantly more likely to become pregnant (70% vs 61%; aOR 1.6, 95%CI [1.2, 2.2]) as well as achieve live birth (51% vs. 39%; aOR 1.6, 95%CI [1.2, 2.2]) compared to those in the standard protocol. (See Table 3 for logistic regression results). In multivariable linear regression models, patients undergoing FETs with the surveillance protocol also had a significantly higher implantation rate (*p* = 0.04). (Not shown).

Analyses restricted to freeze-all cycles were similar when comparing live birth rates between the surveillance and standard protocols (aOR 1.6, 95%CI [1.1, 2.5]). We also performed sensitivity analyses stratified by PGT. In patients who did not have PGT, the odds of live birth in the surveillance protocol was still increased (aOR 1.6 95%CI [1.2, 2.3]). In the population of those who underwent PGT, the odds of live birth in the surveillance protocol was similarly increased (aOR 1.5 95%CI [0.8, 2.8]), however it did not reach statistical significance. Given the possibility that women may have had embryos frozen via the slow-freeze method prior to transfer during the study period, an additional restricted analysis was performed removing all women with embryo cryopreservation performed prior to adaption of the vitrification protocol. In total, 38 women were removed and results for both primary outcomes of pregnancy and live birth remained unchanged.

### Baseline characteristics: those with versus without dose adjustments in surveillance protocol

Women in the surveillance protocol whose hormone doses were adjusted based on surveillance labs were not significantly different from those without dose adjustment in terms of age, endometrial thickness, infertility diagnosis, use of PGT or number of embryos transferred. Mean BMI was significantly higher in the dose adjusted group compared to the dose unadjusted group. (*p* < 0.001) (See Table 2) During the surveillance protocol time period, progesterone dosage was increased in 26% of cycles, estradiol in 11%, and both in 33%. Obese women were more likely to have lower progesterone levels requiring dose adjustment (OR 3.2, 95%CI [2.0, 5.3]). Of the women who underwent PGT in the surveillance protocol, 28% required hormone dose changes and 72% did not.

**Table 2:** Baseline Characteristics of those whose treatment was changed due to pre-transfer surveillance labs versus those where labs were measured but treatment was not changed

	Patients with change in hormones (***N*** = 175)	Patients without change in hormones (***N*** = 349)	***p-***value
**Mean Age (years)**	34.2 (+ 4.8)	34.8 (+ 4.1)	0.2
**Mean BMI (kg/m**^**2**^**)**	26.8 (+ 5.8)	23.6 (+ 4.8)	< 0.001
**Endometrial stripe (mm)**	10 (+ 2.4)	9.6 (+ 2.1)	0.06
**Infertility Diagnosis (%)**	Unexplained – (23%)	Unexplained – (27%)	0.3
	DOR – (3%)	DOR – (7%)	
	Endometriosis – (5%)	Endometriosis – (3%)	
	Ovulation disorders – (11%)	Ovulation disorders – (10%)	
	Male factor – (25%)	Male factor – (19%)	
	Other^a^ – (33%)	Other^a^ – (34%)	
**Number of embryos transferred per cycle, n (%)**	1–118 (67.4%)	1–233 (67%)	0.8
	2–56 (32%)	2–112 (32%)	
	3–1 (0.6%)	3–4 (1%)	
**PGT testing of embryo, n (%)**	46/175 (26%)	121/349 (35%)	0.05

^a^ = Included patients with tubal factor, uterine factor and other as infertility diagnoses

### Dose adjusted versus dose unadjusted cycles in surveillance protocol

In multivariable logistic regression analyses, those whose dose was adjusted in the surveillance protocol group were as likely to become pregnant as those whose dose did not need to be adjusted (72% vs 66%; aOR 0.8, 95% CI [0.5, 1.2]). They were also as likely to achieve live birth compared to those whose dose was unadjusted (47% vs. 53%; aOR 0.8, 95%CI [0.6, 1.2]) (See Table 3 for logistic regression results). Sensitivity analyses restricted to either solely estrogen dose changes or progesterone dose changes did not change livebirth results substantially (aOR 0.6, 95% CI [0.4, 1.2] and aOR 0.9, 95%CI [0.6, 1.4] respectively) when compared to the overall results in which either medication was changed. In multivariable linear regression models, patients undergoing FETs with dose adjustment were as likely to implant as those without dose adjustment (*p* = 0.3). (Not shown).

**Table 3:** Pregnancy Outcome of Patients in Surveillance vs Standard protocols & Change in Hormones vs No Change

	Surveillance protocol (***N*** = 524)	Standard protocol (***N*** = 379)	aOR (95% CI)	***p***-value	Patients with change in hormones (***N*** = 175)	Patients without change in hormones (***N*** = 349)	aOR (95% CI)	***p***-value
**Positive hCG**^**a**^	366/524 (70%)	231/379 (61%)	1.6 (1.2, 2.2)	0.002	115/175 (66%)	251/349 (72%)	0.8 (0.5, 1.2)	0.3
**Live birth**	268 (51%)	149 (39%)	1.6 (1.2, 2.2)	0.001	83 (47%)	185 (53%)	0.8 (0.6, 1.2)	0.4
**Spontaneous abortion**	51 (10%)	38 (10%)	1.0 (0.7, 1.7)	0.9	13 (7%)	38 (11%)	0.6 (0.3, 1.2)	0.2
**Therapeutic abortion**	4 (0.8%)	2 (0.5%)	1.4 (0.2, 8.1)	0.7	1 (0.6%)	3 (0.9%)	0.7 (0.1, 7.4)	0.8
**Stillborn (> 20 weeks)**	2 (0.4%)	2 (0.5%)	0.9 (0.1, 6.6)	0.9	1 (0.6%)	1 (0.3%)	2.0 (0.1, 35.0)	0.6
**Ectopic Pregnancy**	1 (0.2%)	2 (0.5%)	0.5 (0.1, 6.2)	0.6	0 (0%)	1 (0.3%)	–	–
**Biochemical Pregnancy**	40 (7.6%)	38 (10%)	0.8 (0.5, 1.3)	0.4	17 (10%)	23 (7%)	1.7 (0.9, 3.4)	0.1

All analyses were performed using logistic regression adjusted for age, BMI, number of embryos transferred and PGT

^a^ = Positive hCG represents summation of all pregnancy outcomes listed below this row

## Discussion

We found that pregnancy and live birth rates were significantly higher when implementing the surveillance protocol as compared to the standard protocol. The number of studies evaluating the impact of surveillance blood work on outcomes is limited. Unlike our findings, other studies have not demonstrated a consistent association between pre-transfer serum hormone levels and outcome, with one noting that differences in estradiol concentrations prior to FET did not correlate with clinical pregnancy rates [22]. While we did not have a group of patients with surveillance revealing low estradiol levels but no dose adjustment to compare to (as all patients in the surveillance protocol with inadequate levels had dose adjustments), comparison with patients in the standard protocol would indicate that assessment appears to impact outcome.

Interestingly however, women whose hormone doses were adjusted based on the surveillance labs were as likely to conceive as women whose doses were unadjusted. Although it seems counterintuitive that dose adjustment did not improve rates, we believe this may be related to group characteristics. The group requiring dose adjustment may represent poorer prognosis patients. Interestingly, the mean BMI of women in this group was in the overweight range (mean BMI 26.8 kg/m^2^) compared to the normal BMI in the unadjusted dose group (mean BMI 23.6 kg/m^2^). In addition, 50.6% of women in the dose adjusted group were overweight or obese compared to only 36.6% in the dose unadjusted group. Factors such as obesity have been associated with poorer outcomes [23–26]. It is possible that by adjusting hormone doses, we were able to increase this group’s pregnancy rates to be similar to normal weight, better prognosis patients in the comparison cohort. However, since there was no untreated control group with hormone level monitoring, we acknowledge it is not possible to determine whether this intervention was responsible for the rise in pregnancy rates or by how much. In addition, a sensitivity analysis evaluating pregnancy and live births restricted to non-obese women in the hormone adjusted group versus not-adjusted group within the surveillance protocol cohort also did not show differences in outcomes (not shown). However, as this was not the primary objective, it is difficult to know whether this is an issue of power or if alternative explanations are necessary.

During the timeframe of the standard protocol, PGT was not as common and embryo transfer guidelines were less stringent [27, 28]. Therefore, it is not surprising that women in this group were significantly different with respect to these parameters. In the final multivariate analysis, we adjusted for PGT and number of embryos transferred to account for these differences. It is less likely that results shown are significantly affected by differences in PGT alone as sensitivity analyses yielded similar findings, with the non-significant results likely related to the smaller size of the PGT group.

Our study has several notable strengths. We were able to perform a large retrospective study of women representative of the modern infertile population. Our results remained robust in analyses restricted to freeze-all cycles which generally represent better prognostic patients. However, there are some limitations owing to the retrospective nature. We did not have a group of patients who had pre-transfer lab assessment indicating suboptimal levels but without dose adjustment. Therefore, it is not possible to directly assess how pregnancy rates were influenced by adjusting doses. While it is possible for changes in lab practices between 2013 and 2017 to influence outcomes, overall our management has remained similar, including methods of IVF stimulation and embryo transfer procedure that would most directly impact outcome. For example, the brand of transfer catheters did not change, the catheter loading process remained consistent and the culture system in the laboratory as well as general embryology techniques were not altered. While the gas delivery system to incubators was upgraded from Teflon tubing to copper pipping in July 2016, thawed embryos remain in culture for only approximately 2 h prior to embryo transfer; therefore, we do not feel this would have a significant impact on FET outcomes. In addition, the two cohorts were similar in several pertinent ways including age, BMI and infertility diagnoses. Factors that would have changed with time in laboratory practices, such as prevalence of PGT and number of embryos transferred, were controlled for in all models. In addition, restricted analyses removing those with embryos frozen via the slow-freeze method also showed the same results. We did not evaluate hormone levels after FETs to assess whether levels were still suboptimal; therefore, it is possible that inadequate dose adjustment could contribute to lack of differences. Embryo morphology data was also not analyzed.

In conclusion, we found that pregnancy and live birth rates significantly improved after implementing the surveillance protocol, however rates were not significantly different in those whose dose was adjusted based on surveillance labs compared to those whose dose was not adjusted. We hypothesize that patients with low levels on pre-transfer labs may represent a poor prognosis group, such as obese individuals, and we were able to improve their pregnancy rates to match those who did not require hormonal medication dose adjustment by hormonal testing and subsequent medication adjustment. Given the increased prevalence of FETs, understanding how serum evaluation influences outcome is essential. Based on our findings, pre-transfer or day of transfer serum evaluation is warranted and it is possible that obese women should be prescribed higher doses of progesterone in order to prevent suboptimal levels on pre-transfer blood work. However, data on appropriate titration of intramuscular progesterone by BMI is lacking. Performing blood work the day of transfer immediately prior to transfer may decrease repetitive hospital visits. Prospective randomized control trials are needed to explore this area further.

## Data Availability

All data generated or analyzed during this study are included in this published article.
